# Which communication technology is effective for promoting reproductive health? Television, radio, and mobile phones in sub-Saharan Africa

**DOI:** 10.1371/journal.pone.0272501

**Published:** 2022-08-17

**Authors:** Francesco Iacoella, Franziska Gassmann, Nyasha Tirivayi

**Affiliations:** 1 UNU-MERIT, Maastricht, Netherlands; 2 UNU-MERIT and Maastricht University, Maastricht, Netherlands; 3 UNICEF Office of Research—Innocenti, Florence, Italy; Johns Hopkins University Bloomberg School of Public Health, UNITED STATES

## Abstract

The use of radio and television as means to spread reproductive health awareness in Sub-Saharan Africa has been extensive, and its impacts significant. More recently, other means of communication, such as mobile phones, have received the attention of researchers and policy makers as health communication tools. However, evidence on which of the two types of communication (i.e. passive communication from TV/radio, or active communication through phones) is more effective in fostering better reproductive health choices is sparse. This study aims to identify the potential influence of TV or radio ownership as opposed to cell phone ownership on contraceptive use and access to maternal healthcare. Cross-sectional, individual analysis from eleven high-maternal mortality Sub-Saharan African countries is conducted. A total of 78,000 women in union are included in the analysis. Results indicate that ownership of TV or radio is more weakly correlated to better outcomes than mobile phone ownership is. Results are stronger for lower educated women and robust across all levels of wealth. Interestingly, the study also finds that decision-making power is a relevant mediator of cell phone ownership on contraceptive use, but not on maternal healthcare access. A key takeaway from the study is that, while the role of television and radio appears to have diminished in recent years, mobile phones have become a key tool for empowerment and behavioural change among Sub-Saharan African women. Health communication policies should be designed to take into account the now prominent role of mobile phones in affecting health behaviours.

## 1. Introduction

Women in Sub-Saharan Africa bear a heavy burden when it comes to unmet needs for reproductive healthcare. Maternal mortality in Sub-Saharan African countries is the highest in the world [1] and 25% of women in the region lack access to adequate contraceptive methods [2]. While most of the time unsatisfied demands in healthcare and contraception are due to socio-economic and health system characteristics, awareness plays a role and technology can improve access to healthcare [3, 4]. Better health communication, whether through nurse-to-patient interaction [5], peer discussion [6], or mass media campaigns [7, 8], can increase awareness and foster better access to reproductive health care, or prevent the spread of sexually transmissible infections (STIs) [9–11]. Researchers found that caregiver-to-patient and parent-to-child communication, in particular, hold the greatest potential in helping women making informed decisions about their health [12, 13] and fostering contraceptive use [14]. However, these communication venues might be impractical when cultural norms and taboos within families are a barrier to effective communication [12, 15]. In these situations, passive communication technologies, like TV or radio, or active communication technologies, like mobile phones, act as enablers or alternative sources of information, especially for younger people [16, 17]. Promoting reproductive health awareness through TV, radio, and mobile phones means granting broad access to information which could ultimately translate into better reproductive health outcomes. The paper is structured as follows: Section 2 will present a review on the literature on communication technologies and reproductive health. Section 3 will describe in detail the data and the analytical models adopted in this study. Section 4 will present the results from the analysis, while Section 5 will discuss them in light of current literature, and Section 6 will conclude the paper.

## 2. Communication technologies and reproductive health–a review

Information and communication represent essential goods in healthcare. Knowledge-sharing among practitioners, health-care workers, and patients helps increase the quality of service, reducing inefficiencies [18, 19]. Traditional media and recent innovations in information and communication technologies (ICTs) have brought about large improvements in the health sector in high- as well as in low- and middle-income countries. They have fostered knowledge and awareness among practitioners, patients, and the general population by strengthening health communication practices [20].

The role of mass media in health communication has been firmly established in the literature in the past, and the impact of mass media exposure has been registered even in the absence of targeted health-communication campaigns [21]. Traditional media, such as TV or radio, has been proven to increase women’s autonomy and freedom of expression [22] and control over their reproductive life [23] by reducing gender stereotyping through the display of empowered female figures on screen and through radio [24]. These effects are particularly strong in low-literate areas [23, 24]. Evidence from Sub-Saharan Africa shows a consistent association between exposure to traditional media and greater use of contraception and antenatal care [25, 26]. In Burkina Faso, exposure to a radio-based information campaign has been associated with an increase in reproductive healthcare-seeking behaviour [27]. Similarly, evidence from Nigeria found health-related media adverts to be the most common mean of maternal health awareness (It has to be noted that, in approximately 50% of the cases, adverts were retrieved on the internet and not on a TV or a radio) [28]. In order to explain these positive associations, researchers have often resorted to Bandura’s social learning theory [29]. According to Bandura, social learning influences behaviour through either training (e.g. education) or imitation. TV and radio shows, advertisement, and campaigns are able to provide both. The social learning theory has been used to explain the impact of TV and radio shows on family planning behaviour from Tanzania to Mexico [30, 31].

However, in recent years, studies have found that the link between exposure to television and contraceptive use has weakened compared to the past [8, 32]. One study from South Africa suggests that information dissemination through radios is not always beneficial to women in rural settings [33], while evidence from Ethiopia indicates that exposure to mass media has a significant positive effect on contraceptive use, but does not affect reproductive health knowledge nor fertility preferences [34].

Mobile phones, on the other hand, have been gaining momentum as the preferred communication tools for health awareness raising and knowledge diffusion. Researchers have found that they facilitate health worker-to-patient and patient-to-patient communication by reducing communication costs [35, 36]. In Indonesia, midwives’ use of mobile phones increased access to institutional resources and, consequently, increased their knowledge of reproductive health-related topics [37]. Exposure to mobile-based technology has been proven to increase access to delivery assistance and use of contraceptives [38–40]. Such positive effects have been attributed to cell phones’ capacity to increase agency and aspirations by fostering communicative practices outside the household and granting access to resources that challenge women’s own idealized femininity and traditional gender roles, ultimately influencing their decision-making ability over their own health [41, 42]. Additionally, access to social media (usually enabled by mobile phones) mobilises attention and accountability to women’s rights and challenge gender discriminatory practices [43]. Hence, we argue that Bandura’s social learning theory can be extended to mobile phones, given their power to connect people and facilitate knowledge acquisition.

Communication technologies hold the power to change attitudes and behaviour towards contraception and reproductive healthcare. They allow reproductive health-related information to circulate more broadly and strengthen women’s decision making ability. This holds true for both traditional media [23, 24] and for mobile-phones [41, 42]. However, further analysis is needed to understand which communication channel is more effective in promoting better reproductive health, by comparing passive communication tools like TV or radio with mobile phone technologies, which enable passive and active communication.

To develop effective reproductive health communication campaigns and policies for Sub-Saharan Africa, it is important to understand how ownership of different means of communication is related to reproductive health behaviour, and whether mobile phones are more effective in increasing women’s decision-making power over their reproductive health. The aim of this paper is to shed light on this topic by conducting a multi-country comparative analysis of the potential influence of TV/radio and mobile phone ownership on reproductive health behaviours in Sub-Saharan Africa countries.

## 3. Data and methodology

This paper uses data from the Demographic and Health Survey (DHS) Program. To increase the relevance of findings, only Sub-Saharan African countries with a high risk of maternal mortality, as categorised by WHO, are included. The risk of maternal mortality is based on number of maternal deaths per 100,000 live births. As per WHO classification, a country is considered to have high maternal mortality rate when it exceeds 300 deaths per 100,000 [1]. To be able to provide estimates of recent trends, only the latest DHS surveys for each country have been included in this study. The earliest surveys included were conducted in 2015 (Angola, Malawi, and Tanzania) while the latest in 2019 (Sierra Leone). An additional reason for including only most recent DHS survey is because questions on mobile phone ownership have only been included in the questionnaire in recent times. Finally, since the analysis of decision-making power is an essential part of this study and questions on decision-making power are only asked to women in a relationship, only women in union (i.e. either married or with a partner) have been included in the model. The final sample includes 78,000 women aged 15–49 from eleven countries in SSA, nominally: Benin, Burundi, Cameroon, Guinea, Malawi, Mali, Nigeria, Sierra Leone, Tanzania, Uganda, and Zimbabwe. For robustness, analysis of a full sample of women (including those not in union) has been run for the main model and results are reported in Table A1 in the S1 Appendix. Findings are comparable to those of women in union. It is also important to mention that in the analysis of maternal healthcare access, only women who had at least one pregnancy are included (see later in this section).

The explanatory variable of interest is women’s ownership of means of communication. It is operationalised as a categorical variable with three categories: not owning any communication mean, owning a TV or a radio (or both), and owning a mobile phone. Owners of both TV or radio and a mobile phone are therefore excluded from the analysis. With this approach, richer households are potentially excluded from the analysis, although it provides the great advantage of easing comparability between the remaining groups.

Reproductive health outcomes included in the study are use of modern contraceptive methods and, for women who were pregnant at least once (i.e. 74% of the sample), whether they delivered their last child in a safe facility (defined as either a private or public medical facility), and whether they received full antenatal care as defined by the World Health Organisation. Full antenatal care is defined as having received: four antenatal visits; at least two tetanus toxoid injections during pregnancy, or received one tetanus toxoid injection during the pregnancy and at least one in the three years prior to the pregnancy; and received iron and folic acid tablets [44]. Outcomes are coded as binary variables assigning value one to women who use modern contraceptives, have delivered in a safe facility, or have received full antenatal care. All three outcomes refer to reproductive health choices and behaviour, as the interest of this studies lies in identifying which communication technology is more relevant in changing women’s behaviour.

Additionally, the study applies a mediation analysis to understand to what extent the outcomes are the result of women’s decision-making power.

Confounders are added to the analysis to account for potential determinants of reproductive health behaviour and decision-making that co-vary with ownership of communication technologies and might, therefore, bias results. These confounders are selected based on the previous literature on relevant reproductive health determinants [45, 46]. The analysis controls for women’s age, employment status, education, and whether they married before they turned 18. As all women in the sample are in a relationship, partners’ characteristics in the form of partners’ education (i.e. whether they had at least primary education) and employment status are added as confounders as well. Household level characteristics accounting for wealth (an asset wealth score is built as a principal component factor including access to electricity; ownership of at least one refrigerator, bicycle, motorcycle, and/or car or truck; floor material; type of toilet facility; and source of drinking water), gender of the household head, and area of living (i.e. urban or rural) are included as well. Finally, variables are added proxying for healthcare use and related barriers. We include information on whether the respondent visited a health facility in the past 12 months and considers distance or financial resources an obstacle to visiting a health facility.

### Data analysis

A binary model in the form of a probit regression is used to estimate the probability of positive outcomes under the condition of owning a TV or radio or a mobile phone compared to not owning any of the three. The main model is operationalised as follows:

$${RepHealth}_{i}=\beta_{0}+\beta_{1}{TV/radio}_{i}+\beta_{2}{Phone}_{i}+\beta_{3}Z_{i}+\beta_{4}{C+u}_{i}$$ (1)

Where *RepHealth_i_* represents the probability of a woman *i* showing a positive reproductive health indicator. *β*_1_ coefficient estimates the association between owning TV or radio and reproductive health outcomes for each woman *i*, while *β*_2_ estimates effect of owning a mobile phone. Both coefficients are measured against not owning any of the selected communication technologies and are expressed as marginal effects. Lastly, *β*_3_ measures the correlation of covariates Z, while *β*_4_ is a vector of country-fixed-effects. To ensure representativeness, all models are weighted by individual sample weights. Standard errors are clustered at community level to account for survey design. DHS respondents are selected within clusters that usually resemble their community. It is therefore relevant to cluster standard errors at this level to account for socio-economic and cultural similarities between women living in the same cluster.

The main specification might suffer from identification bias as women owning mobile phones might be significantly different in socio-economic characteristics from non-owners. Although we are not able to fully solve this endogeneity problem and claim causal relations, the main findings are confirmed by a decomposition analysis following the Oaxaca-Blinder method [47] and sub-group analysis, which divides the sample according to women’s characteristics, such as education and wealth. Additionally, mediation analysis provides useful insights on the role played by decision-making power in shaping the potential influence of communication technology on reproductive health. This mediation model is estimated following Hayes and Preacher’s (2014) [48] method for mediation analysis with a multi-categorical independent variable. The resulting equation for the mediation analysis is the following:

$${DecPower}_{i}=\gamma_{0}+\gamma_{1}{TV/radio}_{i}+\gamma_{2}{Phone}_{i}+\gamma_{3}Z_{i}+\gamma_{4}C_{i}+u_{i}$$ (2)

$${RepHealth}_{i}=\vartheta_{0}+\vartheta_{1}{TV/radio}_{i}+\vartheta_{2}{Phone}_{i}+\vartheta_{3}{DecPower}_{i}+\vartheta_{4}Z_{i}+\vartheta_{5}C_{i}+\varepsilon_{i}$$

where the indirect (mediated) effect of TV/radio ownership through decision-making power is provided by *γ*_1_*ϑ*_3_, and the indirect effect of mobile phone ownership is provided by *γ*_2_*ϑ*_3_.

For contraceptive use, the mediator is a binary variable with value one for women reporting any decisional power over contraceptive use (i.e. whether she alone, or together with her partner, can decided whether to use contraceptive methods). For access to maternal health care the mediator is a binary variable with value one for women with any decision-making power over their own health (i.e. whether or not they can take decisions over their own health alone or together with their partner). An argument can be made that decision-making power over own health could also be a relevant mediator for modern contraceptive use. For this reason, an alternative mediation analysis of contraceptive use has been run using this decision-making variable instead of decision-making over contraceptive use. Results are discussed in the next section and presented in Table A3 in the S1 Appendix.

## 4. Results

### Descriptive statistics

**Fig 1:**
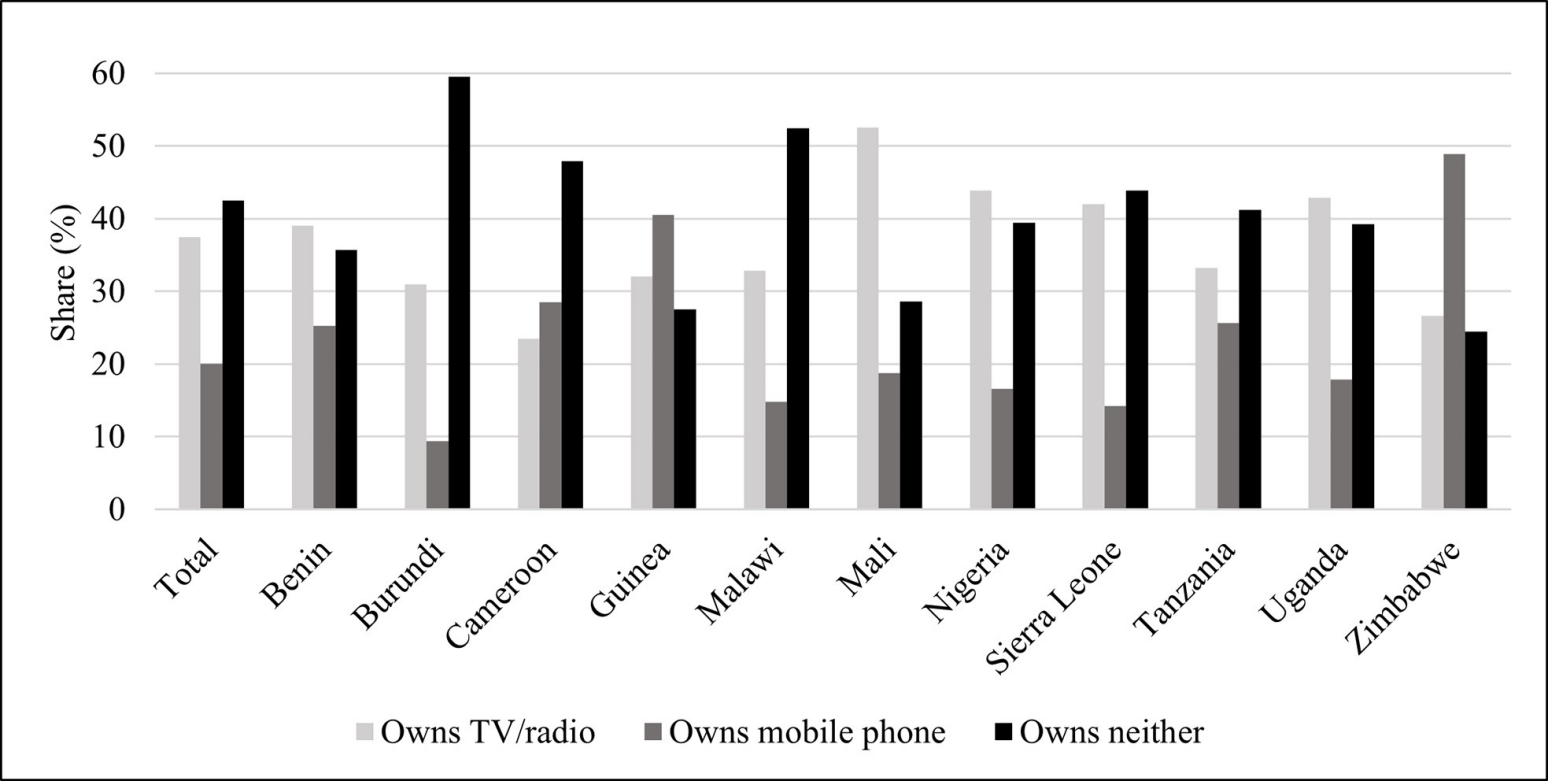
Prevalence of communication means among women.

Fig 1 illustrates communication means’ prevalence within the sample and by country. Overall, approximately 43% of the women in the sample report not owning any of the selected communication means. Burundi (59%), Malawi (52%), and Sierra Leone (44%) report the highest levels of non-ownership. Mali is the country with the highest share of ownership of either TV or radio (54%), followed by Nigeria (45%) and Sierra Leone (43%). Lastly, Zimbabwe (46%), Guinea (37%), and Cameroon (33%) are the only three countries in which women are more likely to own only a mobile phone than only a TV or radio.

Table 1 reports descriptive statistics for outcome and confounding variables in the sample. The average age of sampled women is 30.8. About 74% of women had at least one child, 11% were in union before the age of 18, and 39% of them have a partner who has completed primary education. 72% of sampled women are engaged in paid work and they have on average 3.2 years of completed education. 60% of sampled women are equally distributed between the lowest and the second-lowest wealth quintiles, while only 3% are in the top quintile. This is most probably due to the fact that women owning both TV/radio and mobile phones, potentially the richest ones, are excluded from the main sample (see below for the robustness analysis which also includes those women). About 24% of sampled women declare to be using modern contraceptive methods, although about 81% of them have decision making power over using them. About 62% of sampled women delivered their last child in a safe facility, while 30% received full antenatal care and 51% show complete decision-making power over their own health. 59% of sampled women have visited a health facility in the past 12 months; 83% of them live in a rural setting, and about 14% are in a female-headed household.

**Table 1:** Descriptive statistics of the sample, outcomes, mediators, and confounders.

	Total	Benin (2017)	Burundi (2017)	Cameroon (2018)	Guinea (2018)	Malawi (2015)	Mali (2018)	Nigeria (2018)	Sierra Leone (2019)	Tanzania (2015)	Uganda (2016)	Zimbabwe (2015)
***Sample size***	77,945	6,819	7,847	4,180	3,802	11,943	4,391	16,382	7,052	5,119	7,568	2,848
Use of modern contraceptive (%)	24	11	22	10	9	58	13	7	18	29	31	62
Any decision-making power over contraceptive use (%)	81	79	88	69	74	90	63	76	77	88	86	92
Delivered in a safe facility (%)*	62	79	81	48	43	91	59	23	82	54	69	66
Received full antenatal care (%)*	30	29	9	30	16	36	17	30	70	24	38	34
Any decision-making power on own health (%)	51	42	70	45	39	65	18	30	42	69	71	84
***Individual confounders***												
Woman age	30.8	30.6	32.1	31.8	31.2	29.9	30.2	30.6	32.2	30.5	30	30.1
Woman currently working (%)	72	80	88	73	69	68	56	63	86	79	80	36
Woman years of education	3.2	1.2	2.3	3.3	0.8	4.8	1	2.7	2	5	4.9	8.3
Married when <18yo (%)	11	9.3	6	10	1	13	12	13	9	10	11	10
Partner has primary education (%)	39	17	28	44	13	42	9	43	29	68	49	87
Partner is working (%)	94	98	96	97	93	90	89	95	94	99	96	79
***Household confounders***												
Female-headed HH (%)	14	16	13	12	13	16	13	6	18	11	17	34
Rural household (%)	83	70	94	77	87	91	90	78	78	82	87	82
*Wealth Index quintiles*												
Poorest (%)	30	19	48	43	24	15	21	22	37	58	42	17
Poorer (%)	30	19	41	15	19	63	24	12	19	16	28	18
Middle (%)	24	40	7	22	36	16	33	36	29	14	19	35
Richer (%)	13	20	3	15	17	5	19	23	12	10	9	19
Richest (%)	3	1	<1	3	4	<1	3	6	3	1	1	10
***Health-related confounders***												
Visited a health facility in the past 12 months (%)	59	46	88	49	37	68	42	42	61	68	77	61
Distance to health facility is considered a problem (%)	46	38	35	55	57	60	36	35	55	51	46	47
Health facility is expensive (%)	60	58	68	80	70	57	48	55	77	56	52	53

Note:

*sample includes only women who had at least one child. **Source:** Author’s elaboration from DHS data from women aged 15–49 in union. Population weights are applied.

### Communication technology and reproductive health

This section presents results from the main analysis. They are reported in their integrity in Table 2. A consistent finding throughout the selected outcomes is the lower magnitude of the estimated marginal effects of TV/radio ownership as compared to mobile phone ownership. Owning a TV or a radio increases the probability of using modern contraceptives by 1.4 percentage points, while owning a mobile phone increases it by 2 percentage points. Similarly, owning a TV or a radio increases the probability of having delivered in a safe facility by 1.4 p.p., while owning a mobile phone is associated with a 5 p.p. higher probability. Finally, TV or radio ownership is only weekly correlated to antenatal care, while mobile phones ownership increases the probability of having accessed full antenatal care by 3.2 p.p. As the potential influence of mobile technology could be different for women who were pregnant at least once, compared to those who were not, analysis on contraceptive use is run also for a sample of women who were never pregnant. Results are reported in Table A2 in the S1 Appendix and show a similar result to that of the main analysis.

**Table 2:** Estimated marginal effects of communication technology on the use of contraceptives, antenatal care and safe delivery.

	Use of contraceptive	Antenatal care	Safe delivery
*Baseline value–owning no communication technology*			
**Owns TV/radio**	0.014***	0.008*	0.014***
	[0.004]	[0.005]	[0.005]
**Owns mobile phone**	0.020***	0.032***	0.050***
	[0.005]	[0.007]	[0.007]
Age	0.002***	-0.001***	-0.002***
	[0.000]	[0.000]	[0.000]
Currently working	0.049***	0.033***	0.036***
	[0.004]	[0.006]	[0.006]
Yeas of education	0.008***	0.009***	0.015***
	[0.001]	[0.001]	[0.001]
Married before age 18	0.019***	-0.015**	-0.032***
	[0.006]	[0.007]	[0.006]
Female-headed HH	-0.039***	0.010	0.018***
	[0.005]	[0.007]	[0.007]
Partner’s education	0.028***	0.049***	0.068***
	[0.004]	[0.005]	[0.006]
Partner’s employment	0.012	-0.008	-0.010
	[0.008]	[0.010]	[0.010]
Rural HH	-0.015**	-0.032***	-0.066***
	[0.006]	[0.009]	[0.010]
*Baseline value–poorest*			
Poorer	0.011**	0.007	0.025***
	[0.005]	[0.006]	[0.006]
Middle	0.018***	0.019**	0.058***
	[0.006]	[0.007]	[0.007]
Richer	0.039***	0.045***	0.117***
	[0.007]	[0.010]	[0.010]
Richest	0.060***	0.033*	0.192***
	[0.013]	[0.017]	[0.017]
Visited a health facility	0.044***	0.061***	0.056***
	[0.004]	[0.005]	[0.005]
Problem for visiting–distance	-0.018***	-0.033***	-0.068***
	[0.004]	[0.006]	[0.006]
Problem for visiting—resources	0.008**	-0.005	0.011**
	[0.004]	[0.005]	[0.005]
Country FE	Yes	Yes	Yes
Observations	73,570	53,863	53,863
Pseudo R^2	0.191	0.103	0.276

**Note**: “HH” stands for household. “Problem for visiting–distance” refers to considering distance to health facility a problem. “Problem for visiting–resources” refers to considering financial resources a problem when utilising health facilities. Results are presented as marginal effects. Standard errors are clustered at the community level (in brackets).

*** p<0.01

** p<0.05

* p<0.1.

It is worth noting that all confounders are significantly correlated with the outcomes. The most consistent positive predictors of better reproductive health practices are women and partner’s education, employment status, and frequency of visiting a health facility. Additionally, wealth is a positive and significant predictor of all outcomes.

Given the relevance of our confounders in influencing reproductive health outcomes, it is important to investigate whether systematic differences in these confounders between communication technology owners and non-owners are driving our results. A decomposition analysis of inter-group (i.e. owners and non-owners) differences in outcomes is performed using the Oaxaca-Blinder (OB) method [47]. The OB decomposition is intended to disentangle the effects related to the socio-economic characteristics from effects related to communication technology ownership. The extent to which ownership contributes to the gap in outcomes between owners and non-owners is included in the unexplained component. Results, presented in Table A4 in the S1 Appendix, show how the unexplained component represents between 30% and 110% of the total gap in outcomes, and is always statistically significant. The OB analysis, therefore, confirms that the potential influence of communication technology ownership on reproductive health is non negligible even after baseline characteristics have been accounted for.

As a robustness check, the main analysis presented in Table 2 has been repeated with the inclusion of women owning both TV/radio and mobile phone. Results are presented in Table A5 in the S1 Appendix and confirm the higher magnitude of the marginal effects of mobile phone ownership as compared to TV/radio ownership. The results also show that ownership of both communication technologies is strongly associated with maternal healthcare and contraceptive use (large and statistically significant marginal effects are observed).

### Heterogeneity of results by education and wealth

In order to better understand the role communication technologies can play for women from different socio-economic backgrounds, we analyse them separately. The positive effects of exposure to mass communication are felt particularly in low-literate areas, as communication technology replaces institutional sources of information and knowledge [23, 24]. Moreover, the level of education is an important predictor of health service utilization and outcomes [49, 50]. It is therefore relevant to check whether TV or radio ownership or mobile phone ownership are equally important for women with different educational attainment. Table 3 presents results from Eq (1) estimated separately for women with no education, primary education, or higher-than-primary education. Overall, the association of TV/radio ownership with reproductive health behaviour is weaker than that of mobile phone ownership. For women with no education, TV or radio ownership shows no association to reproductive health behaviour, while for women with primary education, the magnitude of the coefficient is lower than that of mobile phones. Mobile phone ownership has the strongest correlation with contraceptive use for uneducated women, and with safe delivery for women with no or primary education. Hence, using mobile phones as a means of communication may help bridging knowledge gaps and increase the awareness among those women with no or only little formal education experience.

**Table 3:** Estimated marginal effects of communication technology on reproductive health by educational attainment.

	No education			Primary education			Higher-than-primary education		
	*Use of contraceptive*	*Antenatal care*	*Safe delivery*	*Use of contraceptive*	*Antenatal care*	*Safe delivery*	*Use of contraceptive*	*Antenatal care*	*Safe delivery*
**Owns TV/radio**	0.008*	0.002	0.004	0.020***	0.006	0.028***	0.006	0.041**	0.004
	[0.004]	[0.007]	[0.007]	[0.007]	[0.008]	[0.008]	[0.014]	[0.017]	[0.014]
**Owns mobile phone**	0.024***	0.033***	0.050***	0.026***	0.033***	0.052***	-0.003	0.045**	0.036***
	[0.007]	[0.009]	[0.010]	[0.009]	[0.011]	[0.010]	[0.016]	[0.018]	[0.014]
Confounders	Yes	Yes	Yes	Yes	Yes	Yes	Yes	Yes	Yes
Country FE	Yes	Yes	Yes	Yes	Yes	Yes	Yes	Yes	Yes
Observations	36,803	25,969	25,969	26,392	20,022	20,022	10,375	7,872	7,872
Pseudo R^2	0.150	0.133	0.300	0.132	0.064	0.180	0.137	0.054	0.179

**Note**: Results are presented as marginal effects. Standard errors are clustered at the community level (in brackets).

*** p<0.01

** p<0.05

* p<0.1.

**Fig 2:**
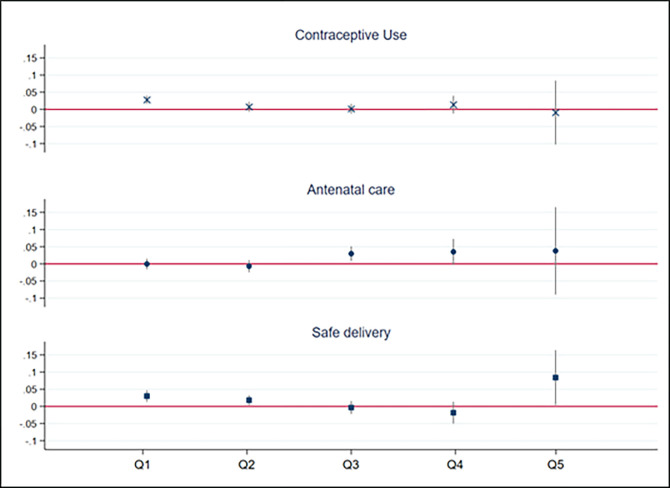
Mobile phone ownership marginal effects on reproductive health by wealth quintile.

The prominent role played by economic status on reproductive health outcomes has been well documented in the literature [45, 51, 52]. Yet, wealth and ownership of means of communication are also positively correlated, as wealthier people can allocate a larger share of their budget to non-food purchases [53]. That begs the question whether the positive correlation between mobile phones and other means of communication and health-related behaviours holds across the different wealth quintiles. Fig 2 depicts the predicted marginal effects of owning a mobile phone for all outcomes disaggregated by wealth quintile. Results remain consistently positive (with the exception of contraceptive use in the richest quintile) regardless of women’s household wealth. Although the size of the coefficients is comparable, results are only significant for lower wealth quintiles. Marginal effects of TV or radio ownership by wealth quintile are reported in Fig A1 in the S1 Appendix and show a similar trend. These findings reinforce the idea that observed results are driven by ownership of communication means and not by overall larger wealth.

### Decision-making power as a mediating factor

The baseline model established the correlation between communication technology ownership and better reproductive health, with mobile phone ownership having a larger potential influence on behaviour than TV or radio ownership. The question is what underlying mechanism is driving these results. We hypothesize that the knowledge obtained through the different means of communication strengthens decision-making agency of the women. Using Hayes and Preacher’s (2014) [48] mediation analysis methodology, we tested whether the findings can be explained by increased decision-making power and present the results in Table 4. Decision-making power over one’s own health or contraceptive use does not appear to mediate the association between TV/radio ownership and reproductive health behaviour. Decision-making power over the use of contraceptives appears to mediate about 37% of the effect of mobile phone ownership on its use, while decision-making power over one’s health does not represent a significant mediator of access to maternal healthcare even for mobile-phone ownership. As a robustness check, the role of decision-making power over one’s health has also been tested as a mediator of contraceptive use. Results, presented in Table A3 in the S1 Appendix show that this represents only a marginally significant mediator of mobile phone ownership.

**Table 4:** Estimated effects of women’s decision-making power mediating the effect of communication technology on reproductive health outcomes.

	Use of contraceptive	Antenatal care	Safe delivery
**Owns TV/radio**			
Indirect effect of decision-making over contraceptive use	-0.003		
	[0.007]		
Indirect effect of decision-making over own health		-0.002	-0.002
		[0.002]	[0.002]
Share of independent effect over total effect	-5.5%	-6.2%	-3.3%
**Owns mobile phone**			
Indirect effect of decision-making over contraceptive use	0.030***		
	[0.008]		
Indirect effect of decision-making over own health		0.001	0.001
		[0.002]	[0.003]
Share of independent effect over total effect	37.1%	1.3%	1%
Confounders	Yes	Yes	Yes
Country FE	Yes	Yes	Yes
Observations	63,213	53,863	53,863

**Note**: Results are presented as percentage points. Standard errors are clustered at the community level (in brackets). *** p<0.01, ** p<0.05, * p<0.1.

## 5. Discussion

This study offered a comprehensive look at the potential influence of TV, radio, and mobile phone ownership on reproductive health choices with regards to contraceptive use and maternal health. It provided evidence from eleven Sub-Saharan African countries of the evolving role played by communication means in recent years, highlighting the importance of mobile phones as a motor of behavioural change in the region.

The first and potentially most relevant result of the analysis is that the association of ownership of TV or radio with contraceptive use or maternal healthcare access is weaker than that of ownership of mobile phones, which is line with findings on the declining effects of exposure to television and radio and contraceptive use [32, 54]. Findings support the idea that the effect of ownership of TV and radio alone on reproductive health outcomes has become less relevant.

On the other hand, mobile phones are consistently correlated with better reproductive health behaviour. This could potentially be explained by mobile phones’ ability to connect individuals more easily and spread knowledge, which are both key promoters of behavioural change according to the social learning theory [29]. Several studies have confirmed the effectiveness of mobile phone-based campaigns to raise contraception and HIV awareness [55, 56]. Evidence is growing in support of a generalised positive impact of mobile phone ownership on reproductive health care, and this study supports these findings [47, 57]. The analysis of heterogeneous effects indicates that less-educated women are those benefitting the most from ownership of mobile phones, as reported by previous studies [58]. Findings are also consistent across wealth quintiles for all indicators, with minor discrepancies for the top quintile. Higher incomes and greater wealth have historically been associated with better health outcomes [59]. However, recent evidence has shown how mobile phone-enabled health communication has been successful in reducing health behaviour gaps for marginalised groups [60]. Findings support this evidence by showing that the positive potential influence of mobile phone ownership for the poorest is statistically significant and may help closing the gap with better-off women.

Mediation analysis looked into possible pathways explaining the positive effects of communication technology ownership on reproductive health behaviour. While no mediation effect of decision-making power is identified for TV/radio ownership, the correlation between mobile phones and a higher probability of using modern contraceptives appears to be mediated by increased decision-making power over contraceptive use for women. The indirect effect of decision-making power accounts for almost 40% of the total effect. A strong correlation between mobile-phone ownership and decision-making over the use of contraceptives, and use of contraceptives itself has been identified by Rotondi et al. (2020) [42]. However, this is the first time, to the authors’ knowledge, that the mediation effect of decision-making power is identified for this topic, supporting the literature theorising the empowering effect of mobile phones [41]. The mediating role of decision-making, however, appears to be limited to contraceptive use, as it does not represent a relevant mediator of the total effect of mobile phone ownership on maternal healthcare access (i.e. full antenatal care and safe delivery). Previous literature identified the low relevance of women’s decision-making power when accessing skilled birth attendance. Even women with high decision autonomy have been shown to rely on family advice when it comes to delivery care [61, 62], and more so in cases of obstetric emergencies [63]. Additionally, cultural norms might make it preferable for some women to deliver at home under the care of traditional birth attendants [64]. Alternatively, women empowerment has usually been associated with better antenatal care [65, 66]. One way to explain the discrepancy between our findings and previous evidence is the fact that we consider full antenatal care, as defined by WHO, as our outcome. Antenatal care is costly, especially for low-income households [67]. Therefore, while decision-making power might have an effect on access to some antenatal care, it might be less relevant in ensuring women get complete treatment.

The present study has some limitations. The nature of the data does not allow for a rigorous assessment of the causal relationship between communication technologies and reproductive health behaviour. The analysis for different population groups and the mediation analysis are used to support and expand the main findings. To be able to compare owners of TV/radio with owners of mobile phones, the analytical framework of this study excludes owners of both assets from the models. While this has clear benefits for the interpretation of results, it also skews the sample significantly towards poorer households (see Table 1). Population weights and various controls are added to the analysis to account for this skewedness, although it has to be taken into consideration when interpreting the external validity of results.

Results from this multi-country study represent a useful starting point for future research focusing on the mechanisms of communication technologies’ impact on reproductive health. Future studies could investigate the role of networks in shaping the effects of mobile phones, an analysis that was not possible for this study due to data limitations. Similarly, the availability of longitudinal data would allow for the analysis of causal inference of the effects of communication technologies on health.

Results also offer suggestions to practitioners and policymakers on how to efficiently shape reproductive health communication policies to achieve behavioural change. National governments can implement policies that make larger use of mobile phones and invest in mobile connectivity infrastructure can help achieve long-lasting results in an ever-more connected Sub-Saharan Africa. National and international actors, even from the public sector, should work together to build platforms that enable individuals to harness the full potential of mobile phones. Audience-centred communication platforms–as in the case of a mobile app aimed at involving fathers in the United States more in their partners’ health choices during pregnancy, can be combined with standard communication campaigns via text messages or hot lines to increase user engagement, resulting in a larger impact on health behaviour [68]. Social media campaigns involving female-role models, who are already leading the way in Sub-Saharan Africa’s digital transformation, can follow in the footsteps of television and radio shows that proved successful in the past [23, 69]. By being exposed to such campaigns, women might feel empowered and “imitate” those role models [30]. Consequently, they might change their reproductive health behaviour following the mechanisms identified in this study.

## 6. Conclusion

This study provides an analysis of the role played by new communication technologies in shaping reproductive health behaviour. While evidence has been collected on whether media exposure or mobile phone ownership influences health practices, this is the first study, to the authors’ knowledge, that compares different communication technologies’ association with reproductive health outcomes over several Sub-Saharan African countries.

While it is important to note that this study cannot prove the existence of a causal relationship, a key takeaway from the findings is that, while the role of television and radio appears to have diminished in recent years, mobile phones have become a key tool for empowerment and behavioural change among Sub-Saharan African women. Findings from this study also shed a light on potential mediators of this association and show that mobile-phone ownership contributes to the empowerment of women to ultimately increase modern contraceptive use, an association that is not shared by TV or radio ownership. On the other hand, decision-making power does not seem to be a relevant mediating factor of safe delivery or full antenatal care access for any communication technology.

## Supporting information

S1 Appendix Contains all the supporting tables and figures. (DOCX)
