# Supplementary material for: Which communication technology is effective for promoting reproductive health? Television, radio, and mobile phones in sub-Saharan Africa
Source: PLoS One. 2022 Aug 17;17(8):e0272501. doi: 10.1371/journal.pone.0272501 (PMC9384982; doi:10.1371/journal.pone.0272501)
Supplement: S1 Appendix — (DOCX) [file pone.0272501.s001.docx]

# **Appendix**

**Table A1.** Marginal effects of communication technology on reproductive health – including women not in union

|  | **Use of contraceptive** | **Antenatal care** | **Safe delivery** | |
| --- | --- | --- | --- | --- |
| *Baseline value – owning no communication technology* | | | |  |
| **Owns TV/radio** | 0.006** | 0.012** | 0.015*** | |
|  | [0.003] | [0.005] | [0.005] | |
| **Owns mobile phone** | 0.044*** | 0.039*** | 0.054*** | |
|  | [0.004] | [0.006] | [0.006] | |
| Age | 0.005*** | -0.001** | -0.002*** | |
|  | [0.000] | [0.000] | [0.000] | |
| Currently working | 0.060*** | 0.033*** | 0.033*** | |
|  | [0.003] | [0.005] | [0.005] | |
| Yeas of education | 0.008*** | 0.011*** | 0.018*** | |
|  | [0.000] | [0.001] | [0.001] | |
| Married before age 18 | 0.079*** | -0.003 | -0.034*** | |
|  | [0.005] | [0.006] | [0.006] | |
| Female-headed HH | -0.055*** | 0.014** | 0.016*** | |
|  | [0.003] | [0.005] | [0.005] | |
| Rural HH | -0.015*** | -0.036*** | -0.061*** | |
|  | [0.005] | [0.008] | [0.009] | |
| *Baseline value - poorest* | | | |  |
| Poorer | 0.011*** | 0.010 | 0.029*** | |
|  | [0.004] | [0.006] | [0.006] | |
| Middle | 0.011** | 0.023*** | 0.065*** | |
|  | [0.005] | [0.007] | [0.006] | |
| Richer | 0.018*** | 0.052*** | 0.123*** | |
|  | [0.005] | [0.009] | [0.009] | |
| Richest | -0.011 | 0.039** | 0.192*** | |
|  | [0.009] | [0.016] | [0.016] | |
| Visited a health facility | 0.069*** | 0.062*** | 0.054*** | |
|  | [0.003] | [0.005] | [0.005] | |
| Problem for visiting - distance | -0.017*** | -0.031*** | -0.065*** | |
|  | [0.003] | [0.005] | [0.005] | |
| Problem for visiting - resources | 0.008*** | -0.006 | 0.007 | |
|  | [0.003] | [0.005] | [0.005] | |
| Country FE | Yes | Yes | Yes | |
| Observations | 116,970 | 62,893 | 63,828 | |
| Pseudo R^2 | 0.163 | 0.100 | 0.262 | |

**Note**: “HH” stands for household. “Problem for visiting – distance” refers to considering distance to health facility a problem. “Problem for visiting – resources” refers to considering financial resources a problem when utilising health facilities. Results are presented as marginal effects. Standard errors are clustered at the community level (in brackets). *** p<0.01, ** p<0.05, * p<0.1.

**Table A2.** Marginal effects of communication technology on contraceptive use – only women who never had a pregnancy

|  | | **Use of contraceptive** | |
| --- | --- | --- | --- |
| *Baseline value – owning no communication technology* | | |  |
| Owns TV/radio | | 0.006* | |
|  |  | [0.003] | |
| Owns mobile phone | | 0.038*** | |
|  |  | [0.004] | |
| Age | | 0.001** | |
|  |  | [0.000] | |
| Currently working | | 0.024*** | |
|  |  | [0.003] | |
| Yeas of education | | 0.006*** | |
|  |  | [0.000] | |
| Married before age 18 | | -0.001 | |
|  |  | [0.016] | |
| Female-headed HH | | 0.010*** | |
|  |  | [0.003] | |
| Rural HH | | 0.006* | |
|  |  | [0.004] | |
| *Baseline value - poorest* |  | |  |
| Poorer | | 0.003 | |
|  |  | [0.005] | |
| Middle | | 0.005 | |
|  |  | [0.004] | |
| Richer | | 0.001 | |
|  |  | [0.005] | |
| Richest | | -0.011* | |
|  |  | [0.007] | |
| Visited a health facility | | 0.010*** | |
|  |  | [0.003] | |
| Problem for visiting - distance | | -0.009*** | |
|  |  | [0.003] | |
| Problem for visiting - resources | | 0.007** | |
|  |  | [0.003] | |
| Country FE | | Yes | |
| Observations | | 32,876 | |
| Pseudo R^2 | | 0.163 | |

**Note**: “HH” stands for household. “Problem for visiting – distance” refers to considering distance to health facility a problem. “Problem for visiting – resources” refers to considering financial resources a problem when utilising health facilities. Results are presented as marginal effects. Standard errors are clustered at the community level (in brackets). *** p<0.01, ** p<0.05, * p<0.1.

**Fig A1**. TV/radio ownership effects on reproductive health by wealth quintile


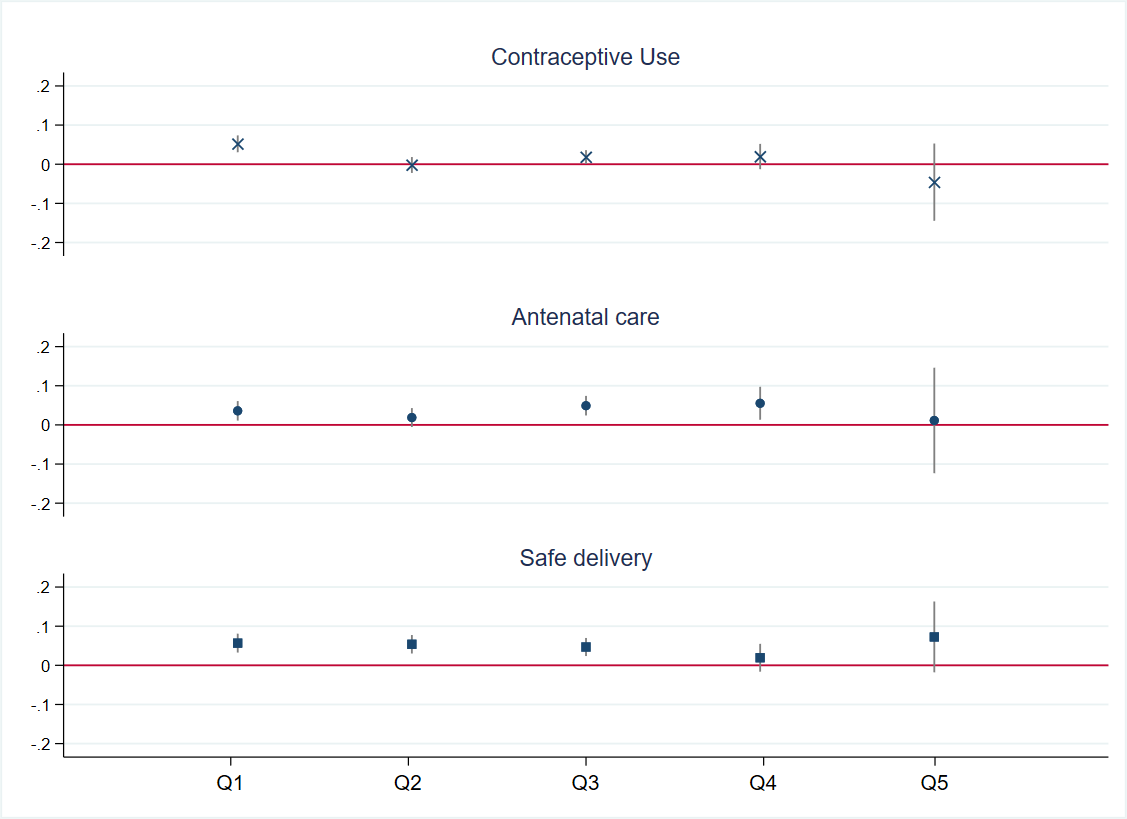


**Note**: “Q1”, “Q2”, “Q3”, “Q4”, and “Q5” represent the five wealth quintiles. Markers represent marginal effects of TV/radio ownership on the selected outcomes. All results are calculated using equation (1). Standard errors are clustered at the community level.

**Table A3.** Decision-making over own health as a mediating factor in the association between communication technology ownership and use of contraceptives

|  | | **Use of contraceptive** | |
| --- | --- | --- | --- |
| **Owns TV/radio** |  | |  |
| Indirect effect of decision-making over own health | | -0.002 | |
|  |  | [0.002] | |
| Share of independent effect over total effect | | -4.4% | |
| **Owns mobile phone** | |  | |
| Indirect effect of decision-making over own health | | 0.005* | |
|  |  | [0.003] | |
| Share of independent effect over total effect | | 6.6% | |
| Confounders | | Yes | |
| Country FE | | Yes | |
| Observations | | 73,569 | |

**Note**: Results are presented as percentage points. Standard errors are clustered at the community level (in brackets). *** p<0.01, ** p<0.05, * p<0.1.

**Table A4.** Decomposition analysis of the differences in reproductive health outcomes between communication technology owners and non-owners

|  | **Use of contraceptive** | **Antenatal care** | **Safe delivery** |
| --- | --- | --- | --- |
|  |  |  |  |
| *Average outcome levels* |  |  |  |
| Communication technology owner | 0.236*** | 0.271*** | 0.596*** |
|  | [0.005] | [0.005] | [0.007] |
| Non-owners | 0.250*** | 0.324*** | 0.634*** |
|  | [0.004] | [0.004] | [0.006] |
| *Difference between groups* |  |  |  |
| Total difference | -0.014*** | -0.053*** | -0.038*** |
|  | [0.004] | [0.005] | [0.006] |
| Explained component | 0.002 | -0.037*** | -0.014*** |
|  | [0.003] | [0.003] | [0.005] |
| Unexplained component | -0.015*** | -0.016*** | -0.024*** |
|  | [0.004] | [0.005] | [0.004] |
|  |  |  |  |
| Observations | 73,570 | 53,863 | 53,863 |
| Confounders | Y | Y | Y |
| Country FE | Y | Y | Y |

**Note**: Communication technology owners includes both TV/radio owners and mobile phone owners Results are presented as percentage points. Standard errors are clustered at the community level (in brackets). *** p<0.01, ** p<0.05, * p<0.1.

**Table A5**. Marginal effects of communication technology on reproductive health outcomes – entire sample

|  | **Use of contraceptive** | **Antenatal care** | **Safe delivery** | |
| --- | --- | --- | --- | --- |
| *Baseline value – owning no communication technology* | | | |  |
| **Owns TV/radio** | 0.014*** | 0.008* | 0.014*** | |
|  | [0.004] | [0.005] | [0.005] | |
| **Owns mobile phone** | 0.020*** | 0.032*** | 0.050*** | |
|  | [0.005] | [0.007] | [0.007] | |
| **Owns both** | 0.040*** | 0.066*** | 0.074*** | |
|  | [0.005] | [0.006] | [0.006] | |
| Confounders | Yes | Yes | Yes | |
| Country FE | Yes | Yes | Yes | |
| Observations | 73,570 | 53,863 | 53,863 | |
| Pseudo R^2 | 0.191 | 0.103 | 0.276 | |

**Note**: “HH” stands for household. “Problem for visiting – distance” refers to considering distance to health facility a problem. “Problem for visiting – resources” refers to considering financial resources a problem when utilising health facilities. Results are presented as marginal effects. Standard errors are clustered at the community level (in brackets). *** p<0.01, ** p<0.05, * p<0.1.
